# Cooperative interactions between p53 and NFκB enhance cell plasticity

**DOI:** 10.18632/oncotarget.2545

**Published:** 2014-10-21

**Authors:** Alessandra Bisio, Judit Zámborszky, Sara Zaccara, Mattia Lion, Toma Tebaldi, Vasundhara Sharma, Ivan Raimondi, Federica Alessandrini, Yari Ciribilli, Alberto Inga

**Affiliations:** ^1^ Laboratory of Transcriptional Networks, Centre for Integrative Biology, CIBIO, University of Trento, Trento, 38123, Italy; ^2^ Laboratory of Translational Genomics, Centre for Integrative Biology, CIBIO, University of Trento, Trento, 38123, Italy; ^3^ Institute of Enzymology, Research Centre for Natural Sciences, Budapest, Hungary; ^4^ Department of Genetics, Massachusetts General Hospital, Boston, MA, USA

**Keywords:** p53, NFkB, chemotherapy, doxorubicin, TNF⍺, EMT, synergy, breast cancer

## Abstract

The p53 and NFκB sequence-specific transcription factors play crucial roles in cell proliferation and survival with critical, even if typically opposite, effects on cancer progression. To investigate a possible crosstalk between p53 and NFκB driven by chemotherapy-induced responses in the context of an inflammatory microenvironment, we performed a proof of concept study using MCF7 cells. Transcriptome analyses upon single or combined treatments with doxorubicin (Doxo, 1.5μM) and the NFκB inducer TNF-alpha (TNF⍺, 5ng/ml) revealed 432 up-regulated (log_2_ FC> 2), and 390 repressed genes (log_2_ FC< -2) for the Doxo+TNF⍺ treatment. 239 up-regulated and 161 repressed genes were synergistically regulated by the double treatment. Annotation and pathway analyses of Doxo+TNF⍺ selectively up-regulated genes indicated strong enrichment for cell migration terms. A panel of genes was examined by qPCR coupled to p53 activation by Doxo, 5-Fluoruracil and Nutlin-3a, or to p53 or NFκB inhibition. Transcriptome data were confirmed for 12 of 15 selected genes and seven (PLK3, LAMP3, ETV7, UNC5B, NTN1, DUSP5, SNAI1) were synergistically up-regulated after Doxo+TNF⍺ and dependent both on p53 and NFκB. Migration assays consistently showed an increase in motility for MCF7 cells upon Doxo+TNF⍺. A signature of 29 Doxo+TNF⍺ highly synergistic genes exhibited prognostic value for luminal breast cancer patients, with adverse outcome correlating with higher relative expression. We propose that the crosstalk between p53 and NFκB can lead to the activation of specific gene expression programs that may impact on cancer phenotypes and potentially modify the efficacy of cancer therapy.

## INTRODUCTION

Cancer cells are continuously exposed to a number of signaling cues that reflect the distinct nature of the microenvironment at primary tumor site, metastastic lesions and potentially also during circulation in the blood stream [1–4]. Therapeutic intervention strategies can result in acute changes in microenvironment signaling, acting also through non-transformed cellular components resident at the primary tumor site [3, 5]. Cellular responses to changes in the microenvironment requires coordinated activation of sequence-specific transcription factors [6], among which NFκB and p53 have a prominent role and often opposing functions [7].

The p53 tumor suppressor gene is activated in response to a large number of cellular stress signals, including genotoxic stress, carbon and oxygen deficiencies, excessive proliferation signals [8, 9]. There are >150 established p53 target genes that link p53 to many different biological outcomes [10–14]. The NFκB family of sequence-specific transcription factors consists of essential regulators of immune, inflammatory, proliferative and apoptotic responses [15], and their activation generally results in the onset of pro-survival signals [16]. The most common form of the NFκB complexes is the p50/RELA (p65) heterodimer. p53 and NFκB activation occurs simultaneously in response to diverse stress conditions, including genotoxic stress and NFκB proteins are frequently de-regulated in cancer, resulting in constitutive activation [17]. Competition between p53 and NFκB for a common limiting cofactor such as p300 can result in mutual inhibition [17, 18]. However, examples of positive interactions have also been reported. For example, it was shown that p65 can induce the p53 target gene p21 by direct binding to its promoter [19] and participates in p53-dependent apoptosis [20]. Several human Toll-like receptors (TLRs), whose signaling leads to NFκB activation [21], were identified as direct p53 target genes both in cancer cells and primary cells [22] and it was demonstrated that p53 and NFκB can cooperate in the activation of pro-inflammatory genes in primary human monocytes and macrophages [23].

To investigate more globally the transcriptional crosstalk between p53 and NFκB we performed a proof of concept study using breast cancer-derived MCF7 cells treated with Doxorubicin, Tumor Necrosis Factor alpha (TNF⍺) and a combination of the two compounds (Doxo+TNF⍺). Our results demonstrated a synergistic interaction between p53 and NFκB transcription factors, which can lead to the reprogramming of cell fate and enhanced migratory potential. Seven genes (PLK3, LAMP3, ETV7, UNC5B, NTN1, DUSP5, SNAI1) were established as synergistically up-regulated after Doxo+TNF⍺ and dependent both on p53 and NFκB. A 29-gene signature of highly synergistic genes up-regulated by Doxo+TNF⍺ appeared to have prognostic value in a cohort of luminal breast cancer patients [24].

## RESULTS

### Striking transcriptome changes upon the combination of Doxorubicin and TNF⍺ treatment of MCF7 cells

We first investigated the potential crosstalk between Doxorubicin (Doxo) and TNF⍺ treatment using gene reporter assays in the human breast adenocarcinoma-derived MCF7 cells (Figure S1A). p53-dependent responsiveness of the P21 and MDM2 promoter plasmid constructs was observed following Doxo treatment and confirmed by p53 silencing. The transactivation of the P21 and MDM2 constructs was reduced upon addition of TNF⍺ to Doxo, suggesting possible inhibition of p53 activity by NFκB. Mutual inhibition of the p53 and p65/RELA proteins has been previously shown on p21 [17], while both inhibition and cooperation were reported at the BAX gene [18, 20]. However, this effect was not observed at the level of the endogenous P21 and MDM2 genes (Figure S1B), which showed similar level of activation in response to either Doxo alone or Doxo+TNF⍺. An NFκB reporter construct was responsive to both Doxo and TNF⍺ as single treatments and showed a strong increase following the double treatment that was unaffected by p53 silencing. On the contrary, the endogenous TNF⍺ and MCP1 NFκB target genes were weakly responsive to Doxo alone, highly induced by TNF⍺ treatment, and showed intermediate induction levels upon double treatment. Hence, canonical p53 or NFκB target genes did not exhibit synergistic transcriptional responses to the combined treatment with doxorubicin and TNF⍺.

**Figure 1:**
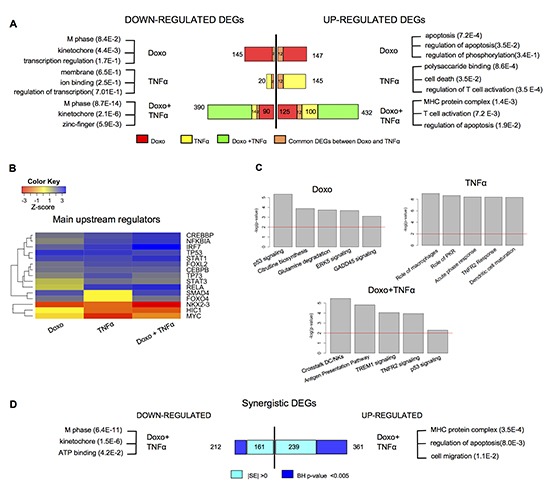
A vast array of genes responds selectively to Doxorubicin and TNF⍺ in MCF7 cells **(A)** Number of DEGs identified after single or combined treatment (see Methods for statistical filters). Most significant gene ontology terms of down- or up-regulated DEGs, according to DAVID (http://david.abcc.ncifcrf.gov). **(B)** Predicted upstream regulators of the DEGs for the indicated treatments, according to IPA (IPA, http://www.ingenuity.com). The color code reflects the enrichment or depletion of the listed transcription factors targeting the DEGs from the array analysis. **(C)** Statistically relevant pathways predicted to be modulated in response to the indicated treatments according to IPA. **(D)** Number of DEGs that are synergistically regulated by the double treatment according to two different statistical filters (see Materials and Methods). The most significant gene ontology terms are also indicated.

Next we performed a genome-wide transcriptome analysis after Doxo, TNF⍺, or the combination of the two compounds using the Agilent 4 × 44k array and single color labeling. Differentially expressed genes (DEGs) were selected based on rank product test, setting a threshold of 0.05 on the percentage of false positives (pfp) and a threshold of 2 on the absolute log2 fold changes. The double treatment more than doubled the number of DEGs (Figure 1). The vast majority of DEGs resulting from the single treatments were also differentially expressed in the double treatment. Gene Ontology (GO) as well as pathway and upstream regulators analyses (DAVID, http://david.abcc.ncifcrf.gov/; IPA, http://www.ingenuity.com/) confirmed activation of p53 signaling upon Doxo treatment as most significant pathway, and apoptosis induction as the most significantly enriched GO terms among up-regulated DEGs (Figure 1A-C). TNF⍺ treatment also resulted in gene annotation terms consistent with NFκB activation, such as regulation of T cell activation. The gene annotation of DEGs resulting from the double treatment was enriched for terms typical of the two single treatments (*e.g.* T cell activation and apoptosis regulation among the up-regulated DEGs). TP53 as an upstream regulator was less significant in the double treatment compared to the Doxo single treatment, while p65/RELA, NFKBIA, IRF7 and STAT1 appeared to be even more enriched in the double treatment compared to TNF⍺ single treatment (Figure 1B). The double treatment not only led to a higher number of DEGs, but resulted in quantitative differences in gene expression levels compared to the single treatments. We applied a rigorous filter and identified 212 repressed, 361 induced DEGs that were synergistically regulated by the double treatment Doxo+TNF⍺ (see Methods) (Figure 1D). Notably, this subgroup of up-regulated DEGs was enriched for cell migration GO biological process along with the expected canonical terms for p53 and NFκB. Collectively, our systematic analysis indicates a vast network of genes that can be mutually affected by combined activation of p53- and NFκB-dependent responses.

### Doxorubicin + TNF⍺ transcriptional synergy identifies new direct p53 and NFκB target genes

We selected fifteen genes for validation experiments based on (a) statistical analysis of synergistic up-regulated DEGs, (b) prior knowledge on direct regulation by either p53 or NFκB, (c) availability of ChIP-seq data for both transcription factors, and (d) gene functions in relation to cancer biology. The selected list contains genes encoding players of the control of various cellular processes, *e.g.* cell proliferation (PLK3, DUSP5, PLAU, GBX2, ETV7, EDN2), apoptosis (TNFRSF10B, UNC5B), inflammation (LAMP3, EGR2), development (GBX2, SOX9, NPPC, FOXC1) and cell migration (SNAI1, PLAU, UNC5B, NTN1, EDN2).

**Figure 2:**
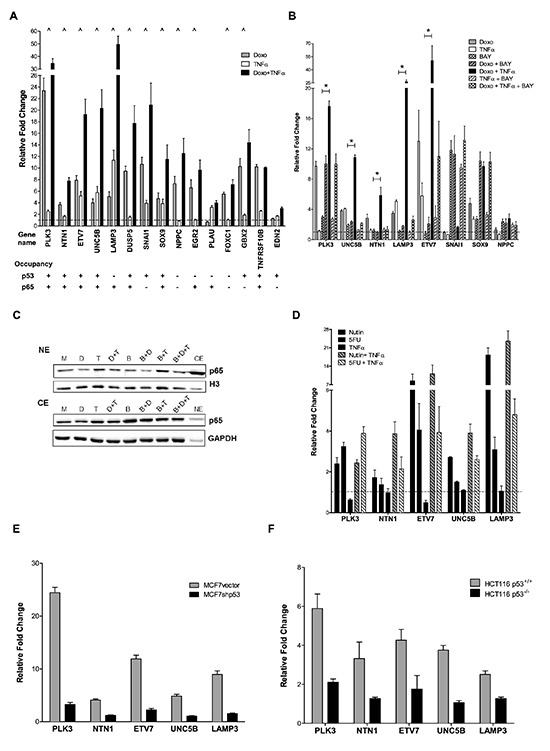
p53- and p65-dependent up-regulation of selected synergistic DEGs **(A)** Twelve out of fifteen selected synergistic DEGs were validated by qPCR. Plotted are the average fold change relative to the mock condition and three reference genes (GAPDH, B2M, ACTB) and the standard deviations of three biological replicates. “^” marks genes responding in synergistic manner to the double treatment. p53 and p65 occupancy data from available ChIP-seq datasets are summarized below each gene name. **(B)** Impact of the NFκB inhibitor BAY 11-7082 on the synergistic gene expression response plotted as in panel A. “*” Significant inhibition of by BAY when combined to Doxo + TNF⍺ (t-test, p<0.01). NPPC and SNAI1 were also tested but their expression levels were not affected by BAY treatment. **(C)** p65 nuclear (NE) and cytoplasmic (CE) relative protein levels under the different treatments used in panel B. M = mock; D = Doxo; T = TNF⍺; B = BAY. Proteins were fractionated as described in Materials and Methods. GAPDH and histone 3 (H3) served as controls for cytoplasmic and nuclear fraction respectively. As controls, a cytoplasmic mock fraction sample (CE) is loaded together with the nuclear proteins and vice versa a nuclear mock sample (NE) in included in the cytoplasmic blot. **(D)** 5-fluorouracil and Nutlin-3a induced expression of 5 selected DEGs alone or in combination with TNF⍺. Results were obtained and are plotted as in A. **(E), (F)** The relative expression of the 5 selected genes shown in panel C was tested in doxorubicin treated matched cell lines differing for p53 status (MCF7 vector and shp53, D; HCT116 p53^+/+^ and p53^−/−^, E).

For twelve of the 15 genes we confirmed a synergistic response to the Doxo+TNF⍺ treatment by qPCR (Figure 2A). Most of them were independently reported as putative targets of either p53, p65 or both according to published ChIP-seq data (for p65, http://genome.ucsc.edu/ENCODE) [14, 25]. A potential direct contribution of NFκB on the observed gene expression changes was evaluates using the small molecule inhibitor BAY 11–7082 (BAY) used as single agent or in combination with Doxo or/and TNF⍺ (Figure 2B). Eight of the twelve validated synergistic DEGs were tested and for five of them BAY markedly inhibited the effect of Doxo+TNF⍺, or of TNF⍺ alone. TNF⍺ treatment led to higher levels of nuclear p65, while Doxo alone or in the combined treatment did not significantly impact p65 nuclear protein levels. BAY treatment led to a slight reduction of p65 nuclear levels, which was paralleled by an increase in the cytoplasm (Figure 2C). p53 protein levels were induced to similar levels by the different treatment combinations (Figure S2).

The five genes that showed more convincing p65 dependence on the synergistic response to Doxo+TNF⍺ (PLK3, NTN1, UNC5B, ETV7, LAMP3) were investigated more deeply to establish a direct role of wild type p53 in their transcription. MCF7 cells were treated with the chemotherapeutic agent 5-Fluorouracil (5FU) or with the MDM2 inhibitor Nutlin-3a, alone or in combination with TNF⍺. Both p53-inducing molecules were at least additive with TNF⍺ in the responsiveness of the five genes (Figure 2D). Although the magnitude of the synergistic response was higher with Doxo, the fact that three different p53-activating treatments led to up-regulation of these five genes strongly suggested a direct role of p53. We next employed an MCF7 clone with stable knock-down of p53 and the HCT116 p53^−/−^ cell line, to further establish p53-dependence of the five genes expression upon Doxo treatment. Matched MCF7 vector and HCT116 p53^+/+^ were used as a comparison (Figure 2E, F). Invariably, Doxo responsiveness was strongly reduced in the p53-defective cells. Previous reports in the literature demonstrated or suggested p53-dependent regulation of PLK3, NTN1 and UNC5B. Our results confirm those findings and establish, for the first time, the possibility of synergistic regulation by NFκB. PLK3, a polo-like kinase, is an important regulator of the cell cycle and it is involved in the control of hypoxia signaling pathway [26]. NTN1 is ligand for both DCC1 and UNC5B receptors whose signaling can potentially modulate p53 activity, impacting on the decision between cell survival and cell death [27]. LAMP3 is a lysosomal membrane associated protein important in dendritic cells and potentially involved in tumor invasion [28], while ETV7 is a transcription factor associated to cell proliferation and tumorigenesis [29].

**Figure 3:**
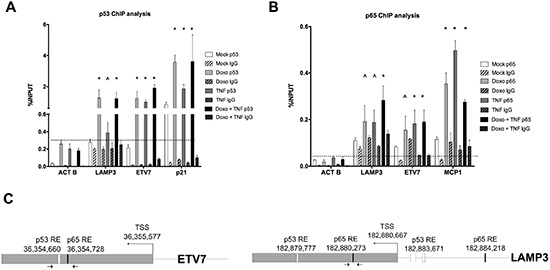
Occupancy analysis establishes ETV7 and LAMP3 as direct p65 and/or p53 target genes **(A)** Relative quantification of immune-precipitated gene fractions by qPCR from MCF7 cells subjected to Doxo or TNF⍺ single treatments and to the double treatment. The antibodies used for the immune-precipitations are listed. P21 was used as positive control, while ACTB was used as a negative control. Plotted are the average percentages relative to input signals. Error bars represent the standard errors of at least three biological replicates. **(B)** as in A, but probing p65 occupancy. MCP1 was used as positive control. The IgG antibody controls were anti-mouse (A) or anti-rabbit (B) to match the specific primary antibodies. **(C)** The position of the primers used for the qPCR and the location of predicted p53 and p65 binding sites in the ETV7 and LAMP3 genes are depicted.

Given the lack of definitive evidence for LAMP3 and ETV7 being direct p53 targets and since our finding of synergistic responsiveness, we examined p53 and p65 occupancy in MCF7 cells treated with Doxo or TNF⍺ (Figure 3). p53 occupancy was detected both for ETV7 and LAMP3 as well as for the positive control P21, in Doxo treated cells. For ETV7 p53 occupancy appeared to increase also after TNF⍺ treatment. P21 was the only target for which p53 appeared to be bound also in the mock condition, a result consistent with previous data [30]. p53 occupancy levels were not distinguishable between Doxo and Doxo+TNF⍺ treatment.

Both LAMP3 and ETV7 exhibited p65 occupancy in TNF⍺ treated cells, although to a lower extent compared to the positive control MCP1. For the three promoter regions, occupancy was increased also by Doxo treatment alone, but no additive effect of the double treatment was apparent, except for a trend with LAMP3. On the contrary lower occupancy at MCP1 was detected in double treated cells. This latter result is consistent with the MCP1 mRNA expression changes (Figure S1B).

Hence, we identified genes whose expression is co-regulated by Doxo and TNF⍺. The gene expression studies conducted with different p53-activating molecules, the use of cells lines with different p53 status, and the chromatin immune-precipitation studies collectively established a direct role for p53 and p65 on the transcriptional regulation of PLK3, NTN1, ETV7, UNC5B and LAMP3. However, we did not find a direct correlation between occupancy levels at predicted promoter binding sites and gene expression changes.

### Doxorubicin + TNF⍺ treatment enhances the migration potential of MCF7 cells

**Figure 4:**
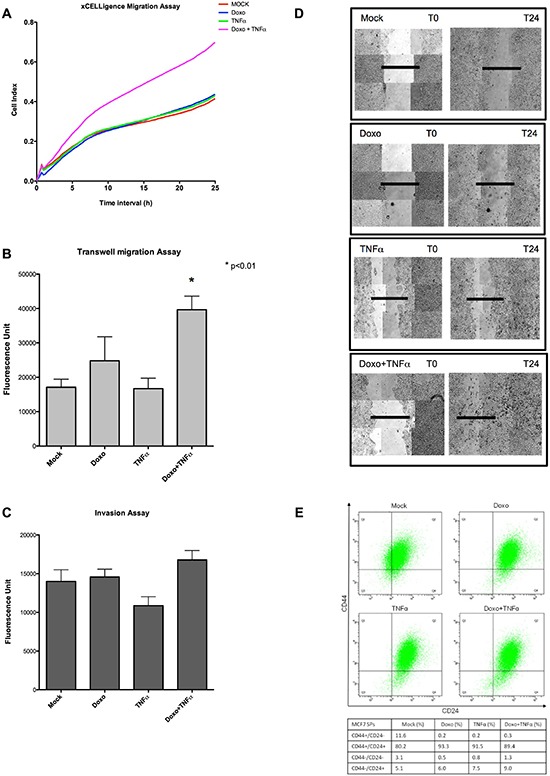
Doxo+TNF⍺ leads to enhanced MCF7 motility but ablates the stem-like side population **(A)** Real-time migration assays examined by xCELLigence. Plotted are the average results of four biological repeats. Cell Index is proportional to the number of cells migrating through a hole in the culture plate. The treatments relative to the different curves are indicated. **(B)** Relative transwell migration values quantified by a fluorescence readout (see Materials and Methods). Average and standard deviation of triplicate biological replicates are presented. The applied treatments are listed on the x-axis. **(C)** As for B, but measuring the invasion potential of MCF7. **(D)** Images of a wound healing assay obtained at T0 or T24. Composite (3×3) images were acquired using an automated Zeiss microscope and the AxioVision3.1 software. **(E)** Cell sorting results based on intensity of CD44 and CD24 surface markers on 30000 cells. Q1 individuates the CD44^+^/CD24^−(low)^ cells, considered as stem-like. The percentages in the four quadrants after the various treatments are presented in the table.

Both the gene ontology enrichments of synergistic DEGs and the known function of the fifteen genes chosen for validation suggested the possible activation of gene expression programs influencing cell motility, epithelial mesenchymal transition (EMT) or even stem-like phenotypes. Projected to an *in vivo* context, the crosstalk of signals present in an inflammatory microenvironment could have a negative impact on the efficacy of chemotherapy, possibly by enhancing tumor cell plasticity. To begin exploring this hypothesis, we investigated migration and invasion potential of MCF7 cells treated with Doxo, TNF⍺ or both. Three different experimental approaches consisting in real-time cell migration analysis (Figure 4A), transwell migration test (Figure 4B) and wound healing assay (Figure 4D) consistently showed higher migration potential of double-treated MCF7 cells, while the invasion phenotype was unaffected by all three types of treatment (Figure 4C).

Several studies suggest that EMT not only enhances the motility and invasiveness of cancer cells, but also provides additional aggressive features such as stemness and therapeutic resistance [31]. Indeed, several of the 15 synergistic DEGs we validated are directly or indirectly associated with acquisition of stem-like phenotypes in normal or cancer cells, particularly SNAI1 [32, 33], SOX9 [34] and GBX2 [35]. Different lines of evidence indicate that breast cancer stem cells (BCSCs) display increased cell motility, invasion, and overexpress genes that promote metastasis [36] and can be traced by CD44^+^/CD24^−/low^ surface marker expression [37]. We asked if the Doxo+TNF⍺ treatment could enhance the stem-like subpopulation of the MCF7 cell line (Figure 4E). FACS analysis showed that the CD44^+^/CD24^−^ subpopulation virtually disappeared after all treatments. Therefore, the higher motility observed upon double treatment cannot be directly related to the expression of these surface markers, hence to putative stem-like features.

### Prognostic value of Doxorubicin + TNF⍺ synergistic DEGs

**Figure 5:**
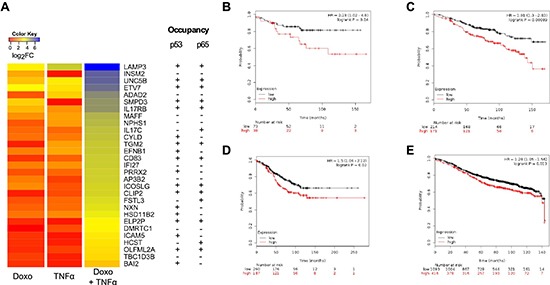
Prognostic significance of a 29-gene list of synergistic Doxo+TNF⍺ DEGs **(A)** Top list of 29 genes (DT-29) exhibiting minimal responsiveness to Doxo or TNF⍺ as single agents, but strong synergy upon combined treatment. A heat map view of the gene expression results is presented (see Materials Methods for statistical filters). Occupancy of both for p65 and p53 in the vicinity of the transcription start sites of these genes has been summarized from ChIP-seq data available in the literature. **(B-E)** Kaplan-Meier plots stratifying a breast cancer patient cohort based on the relative expression of the DT-29 gene list and relapse free survival. Graphs were generated with the KM-plotter tool (ref). Patients' numbers are listed below the graph. Hazardous Ratio and the statistical analysis is reported for selected patients subgroups: **(B)** luminal A patients who underwent chemotherapy treatment (n = 111); **(C)** luminal A patients with a Grade 2 cancer at diagnosis (n = 385); **(D)** luminal A patients with lymph node infiltration at diagnosis (n = 447) and **(E)** the entire cohort of luminal A patients (n = 1509). Patients with a diagnosis of Luminal A breast cancer subtype were selected as the p53 status is not available in KM plotter, but this subgroup of breast cancer is expected to be strongly enriched for cases retaining wild type p53 protein.

Since luminal type breast cancer, of which MCF7 is considered as a model, frequently retains wild type p53 and NFκB responsiveness, we asked if Doxo+TNF⍺ synergistic DEGs could be endowed with prognostic significance. Up-regulated DEGs were further filtered by selecting genes that were strongly responsive to the double treatment but minimally responsive to the single ones (see Materials and Methods). A signature list of 29 genes (DT29) was generated (Figure 5A) and used to interrogate clinical data using the KM plotter tool [38]. Interestingly, breast cancer patients with luminal type A diagnosis who underwent chemotherapy and exhibited higher relative expression of DT29 genes showed poorer prognosis (Figure 5B). The same was true for luminal A patients with lymph node infiltration or luminal A grade 2 (Figure 5C, D).

### Analysis of Doxorubicin and TNF⍺ crosstalk in lung cancer-derived and HUVEC cells

**Figure 6:**
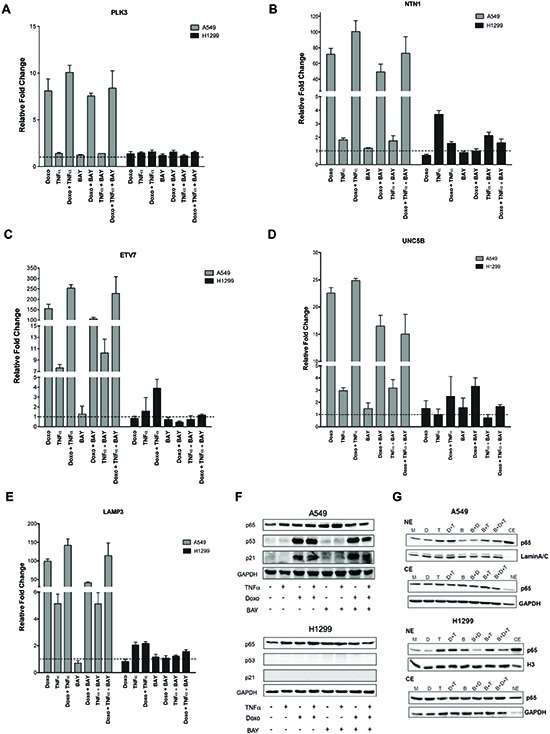
PLK3, NTN1, ETV7, UNC5B and LAMP3 responsiveness in lung cancer cell lines **(A-E)** Relative fold change expression of the indicated genes and after the listed treatments in A549 (p53 wild type) and H1299 (p53 null) cells, measured by qPCR. Average and standard deviations of three biological replicates are presented. **(F)** Western blot of total p65, p53 and the p53 target p21. GAPDH was used as loading control. (G) Western blot of nuclear and cytoplasmic protein fractions were performed as for Figure 2C.

We extended our analysis to another pair of cancer cell lines that differ for p53 status. A549 (p53 wild type) and H1299 (p53 null) lung cancer derived cells were treated with Doxo or/and TNF⍺ or/and BAY. Expression of PLK3, NTN1, ETV7, UNC5B and LAMP3 was measured by qPCR (Figure 6A-E). The impact of the various treatments on p65 nuclear and cytoplasmic, p53 and p21 protein levels was also evaluated (Figure 6F, 6G). In the p53 null H1299 cells the relative expression changes of all the genes was invariably much lower compared to A549 cells. However, NTN1 was weakly TNF⍺ inducible and ETV7 was weakly Doxo+TNF⍺ responsive. Instead in A459 cells NTN1, ETV7 and LAMP3 were synergistically up-regulated by Doxo+TNF⍺, while PLK3 and UNC5B were additive. The magnitude of induction upon Doxo was often one order of magnitude higher compared to TNF⍺ alone. Transient transfection assays with the κB luciferase reporter construct were performed using different concentrations of TNF⍺ or BAY (Figure S3). Based on the results, 10ng/ml TNF⍺ and/or 20μM BAY were chosen for the qPCR experiments, although the reduction of TNF⍺-induced reporter activity was modest, albeit significant. At the endogenous gene level in A549 cells we did not observe the inhibitory effect of BAY on either TNF⍺-induced changes or Doxo+TNF⍺, with the possible exception of UNC5B (Figure 6A-E). However, BAY treatment reduced the Doxo responsiveness of these genes, which might be dependent on its effect on the activation of NFκB by endogenous production of TNF⍺. In the p53 wild type A549 cells, p53 and p21 protein levels were induced by Doxo and not affected by the treatment with TNF⍺. Total p65 levels were unaffected by all treatments in both cell lines (Figure 6F). Nuclear p65 protein levels were increased in response to TNF⍺ or Doxo+TNF⍺ in both A549 and H1299 cells (Figure 6G). Interestingly, BAY treatment alone or in combination led to a reduction in p65 nuclear accumulation (Figure 6G).

HUVEC primary cells were also subjected to Doxo and TNF⍺ single or double treatment and the expression of the same panel of five genes was tested by qPCR (Figure S4). Results among biological repeats varied, but in the majority of tests, all genes with the exception of LAMP3 were Doxo responsive; NTN1 and ETV7 were also TNF⍺ responsive. No synergistic up-regulation by the double treatment could be consistently established. p53 and p65 protein levels confirmed i) the activation of p53, with a similar level of p53 protein in the double treatment, and ii) the p65 proficiency of this cell line.

## DISCUSSION

Wild type p53 functions are intricately related to multiple tumor suppressor pathways, primarily acting in cell autonomous manner to restrain cell proliferation and including cell death and senescence in response to genotoxic and many other types of cellular stresses [8, 9]. Furthermore, p53 also contributes to modulate the microenvironment in a non-cell autonomous manner [39]. p53 has also been linked to inhibition of EMT, for example through an indirect stimulation of E-cadherin expression [40]. At the same time, paracrine signaling in mice triggered by Doxorubicin were found to stimulate EMT and metastatic potential of cancer cells, in part through NFκB activation [3]. Many studies have highlighted the potential contribution of NFκB-induced signaling in the acquisition of cancer cell traits conducive to chemoresistance and higher metastasis risk [2] [41]. While, the canonical functions of p53 and NFκB are consistent with the co-occurrence of p53 inactivation and NFκB hyper-activation that is frequent in cancer [7], recent studies provided examples of positive cooperation between p53 and NFκB that would occur in specific cell types, such as antigen presenting cells or macrophages, and contribute to physiological responses, such as for example in the process of innate immunity and inflammation [12, 22, 23, 42].

Here we modeled the impact of a first line chemotherapeutic drug leading to genotoxic stress and p53 activation, using exposure to the immune cytokine and NFκB activator molecule TNF⍺ as a variable, mimicking the effect of an inflammatory microenvironment. We used transcriptome analysis as primary endpoint and uncovered a vast network of differentially expressed genes that selectively responds to combined treatment with Doxorubicin and TNF⍺. Furthermore, genes that were synergistically up-regulated by both treatments appeared to endow cells with higher motility potential *in vitro*. Analyses of the annotated gene functions related to the aforementioned genes also revealed the possibility of an induced epithelial mesenchymal transition upon combination of the treatments. For example, SNAI1 appeared to be regulated in more than additive manner by the double treatment, as well as LAMP3, a lysosomal protein previously associated with metastasis risk [28, 43]. Multiple cytokines and secreted factors, including IL6, IL17, IL15 and its receptor, S100A8 and S100A9, CXCL12 and several Serpins were also identified as synergistic DEGs (Table S1). The presence of S100A8, S100A9 and CXCL12 among synergistic DEGs raises the possibility that, unlike the case of the triple negative cell line MDA-MB-231 for which S100A8-mediated signaling appeared to require heterotypic cell interactions [3] contributing to metastasis potential, in MCF7 cells this signaling could become homotypic or even autocrine. A marked difference in secreted factors and associated signaling among MDA and MCF7 cells was elegantly shown in recent studies [4].

A direct contribution of p65/RELA and p53 in the observed gene expression changes elicited by Doxorubicin and TNF⍺ was inferred for some of the synergistic DEGs by modulating pharmacologically or genetically p65 or p53 activities. However, we cannot exclude at this stage a (Doxo+TNF⍺)-dependent, but p53- or NFκB- independent gene expression changes. For example, NFkB can functionally interact with AP-1 [44–46] or ER [47], which in turn can modulate p53-dependent responses [48] [49] [50].

Among the most synergistic genes, 29 appear to be prognostic in luminal A breast cancer patients who underwent chemotherapy, where their higher expression correlated with adverse outcome. The majority of luminal A breast cancers are wild type for p53 [51], although data is not available to stratify patients for p53 status in the KM plotter tool [24]. Based on available ChIP-seq data [14, 25, 52, 53], 20 of these 29 genes are putative targets of either p53 or p65 and 10 of them are putative targets of both factors (Figure 5). This result raises the possibility of an unexpected negative outcome of chemotherapy in the context of an inflammatory microenvironment. The prognostic significance of this gene signature needs in-depth evaluation in independent patients cohorts. If confirmed, the results would further support the value of combining treatments activating p53 and repressing NFκB [7].

Given that the crosstalk between Doxorubicin and TNF⍺ and the interplay between p53 and NFκB would occur in cells residing or infiltrating the tumor microenvironment, the ultimate *in vivo* outcome of these functional interactions may vary and cannot be directly predicted from our study using a pure culture of MCF7 cells *in vitro*. Here we have explored Doxo+TNF⍺ impact on HUVEC cells and also on a p53 wild type lung adenocarcinoma-derived cancer cell line. Although limited by the number of genes tested, the results suggest that a positive crosstalk between Doxorubicin and TNF⍺ can be a general characteristic of different cell types and is at least in part p53-dependent, based on the results with a p53 null lung cancer cell line. Furthermore, while we have addressed here the functional interactions between two small molecules, cells are constantly exposed to a complex milieu of signaling factors. However, both p53 and NFκB are master regulators, often contributing a dominant trait in gene expression changes to their target genes. Nuclear receptors, including Estrogen Receptors (ERs) can also modulate NFκB as well as p53 functions [54–56] and have critical roles in breast cancer etiology. We also explored the impact of ER function in the transcriptional programs responding to Doxorubicin and TNF⍺ exposure, using estrogen-depleted culture conditions and adding 17β-estradiol (10^−9^M, E2) as variable (Table S2 and GSE 24065). However, the combination of E2 to Doxo and TNF⍺ resulted only in 15 and 11 selective up- and down-regulated DEGs, respectively (Table S3). A hierarchical cluster analysis of all the treatments confirmed graphically the large difference between TNF⍺- and Doxo-induced transcriptomes and also the significant impact of TNF⍺ when combined to Doxo, while E2 had a minor effect both in the combination with Doxo and with Doxo + TNF⍺ (Figure S5).

With this study we established an example of positive cooperation between p53 and NFκB, in the context of the responses of an epithelial cancer cell to standard chemotherapy but in the presence of active signaling by a pleiotropic inflammatory cytokine, such as TNF⍺. A signature gene of the consequent transcriptional reprogramming appears to be prognostic in breast cancer patients. Associated gene functions indicate the potential acquisition of enhanced cell plasticity and motility and provide a rationale to investigating mechanisms resulting in acquired chemoresistance, particularly for luminal A breast cancer, but potentially with general implication for p53 wild type tumors of different tissue types, and for overcoming such resistance by targeting NFκB. The unexpected positive crosstalk between p53 and NFκB emerging from our and other very recent studies [23] may represent an evolutionary consequence of anti-viral and infection responses towards which NFκB is an established master regulator [57], but the p53 and p73 family member are emerging as important/critical contributors [42, 58, 59].

## MATERIALS AND METHODS

### Cell lines and culture conditions

MCF7 (p53 wild type, expressing p65 and positive for ERs) and HUVEC (Human Umbilical Vein Endothelial Cells) cells were obtained from ICLC (Genoa, Italy), while A549 from ATCC (Manassas, VA, USA). H1299 cells were a gift of Dr. Resnick's laboratory (NIEHS, NIH, RTP, NC, USA); HCT116 p53^+/+^ and p53^−/−^ of Dr. Vogelstein's (John Hopkins Kimmel Cancer Center, Baltimore, MD, USA). MCF7-shp53 or control MCF7-vector cells were provided by Dr. Agami (Netherlands Cancer Institute, Amsterdam, The Netherlands). Cells were cultured in DMEM or RPMI media supplemented with 10% FBS, or Medium 199 (Lonza Milan, Italy) supplemented with 50 units/ml Low Serum Growth Supplements (Life Technologies, Milan, Italy) in the case of HUVEC cells that were also cultured on 0.1% gelatin pre-coated plastics. Media were supplemented by 2mM L-Glutamine and 1XPenicillin/Streptomycin mixture (Pen/Strep), and Puromycin (0.5 μg/mL) in the case of MCF7-shp53 and –vector cells. When appropriate, cells were maintained in DMEM without Phenol Red (Lonza) supplemented with Charcoal/Dextran treated FBS (Hyclone, GE Healthcare, South Logan, UT, USA).

### Drug treatments

Doxorubicin (Doxo, 1.5 μM), 5-Fluorouracil (5FU, 375 μM), Nutlin-3a (10 μM) were used to stabilize p53 protein. When needed TNF⍺ (5ng/ml in MCF7 and 10ng/ml in H1299, A549 and HUVEC cells –based on dose-response tests with gene reporter assays) or BAY11-7082 (10μM or 20μM in H1299 and A549) were added to the culture medium. All compounds were from Sigma-Aldrich (Milan, Italy).

### Microarray experiment and data analysis

Total RNA was extracted from 4 biological replicates using the Agilent Total RNA Isolation Mini Kit (Agilent Technologies, Santa Clara, CA, USA). Samples with RNA Integrity Number (RIN) above 9 (Agilent 2100 BioAnalyzer) were processed. Details are provided with the Gene Expression Omnibus (GEO) (www.ncbi.nlm.nih.gov/geo) submission (GSE24065) and in [56]. The output of Feature Extraction (Agilent standard protocol GE1_107_Sep09) was analyzed with the R software for statistical computing and the Bioconductor library of biostatistical packages. Probes with low signals were removed in order to filter out the unexpressed genes and keep only probes with acceptable signals in most of the replicates. Signal intensities across arrays were normalized by quantile normalization. Signal intensities from probes associated with the same gene were averaged. This procedure resulted in quantitative signals for 14095 HGNC genes. To identify potential target genes of Doxorubicin and TNF⍺, we compared the signals after the double treatment (Doxo+TNF⍺) and the two single treatments relative to the untreated control (mock). DEGs were selected applying a statistical test based on rank products implemented in RankProd Bioconductor package, setting a threshold of 0.05 on the percentage of false positives (pfp) and a threshold of 2 on the absolute log2 fold changes [60]. Every treatment was compared to the mock condition (Table S1, S2 and Figure S5).

To select genes with synergistic effect, i.e. genes whose expression variations were more than additive in the double treatment with respect to single treatments, a further comparison between the double treatment samples and all the remaining samples (single treatments and control samples) was performed (double treatment vs all). Synergistic DEGs were selected applying an additional pfp filter (pfp<0.005) derived from this comparison, to the list of DEGs resulting from the “double treatment vs mock” comparison. A more stringent criterion was obtained by calculating the synergistic effect (SE) of the double treatment as the observed difference between the fold change of the double treatment and the sum of the fold changes of the single treatments (SE=log2 FC double treatment – (log2 FC Doxorubicin + log2 FC TNF⍺). We filtered genes with SE>0 for up-regulated DEGs, SE<0 for down-regulated genes (Figure 1). To select genes where the up-regulation contribution of each single treatment was low respect to the up-regulation of the double treatment, the ratio of the single/double treatments was calculated, applying a 0.25 filter on them (FC Doxorubicin/FC double treatment <0.25 and FC TNF⍺/FC double treatment <0.25) (see Table S1, S2).

### RNA isolation and quantitative qPCR

Total RNA was extracted using Qiagen RNeasy Kit (Qiagen). cDNA was converted from 1 μg of RNA using M-MuLV reverse transcritptase and RevertAid cDNA Synthesis kit (ThermoFisher, Milan, Italy). qPCR was performed on a Bio-Rad CFX384 (Bio-Rad, Milan, Italy). TaqMan gene expression assays (Applied Biosystems, Life Technologies) and Probe MasterMix (Kapa Biosystems, Resnova, Rome, Italy) were used starting with 25ng of cDNA as previously described [56, 61]. GAPDH, B2M or ACTB served as reference genes.

### Western blot

Protein extraction and immunodetections were performed as previously described [62], using ECL Select detection reagent (GE Healthcare) and anti-p53 (DO-1) anti-RelA/p65 (C-20) anti- p21 (C19), anti-GAPDH (6C5) (Santa Cruz Biotechnology, Heidelberg, Germany). When appropriate, nuclear and cytoplasmic fractionation was performed. MCF7, A549 and H1299 cell lines were seeded on 100mm Petri dishes and treated at 80% confluence with Doxo, TNF⍺, BAY or the combination of the drugs for 16 hours. Cells were harvested and cytoplasmic and nuclear proteins were extracted using NE-PER Nuclear and Cytoplasmic Extraction Kit (Pierce, ThermoFisher Scientific), following the instructions provided by the manufacturer. 20 μg of nuclear and cytoplasmic extracts were loaded on a 12% poly-acrylamide gel and transferred to nitrocellulose membranes. Antibodies used for detection were: anti-Histone H3 (clone #: ab1791, AbCam, Milan, Italy) and anti-Lamin A/C (clone #: 2032, Cell Signaling, Milan, Italy) used as nuclear loading control, and anti-GAPDH used as cytoplasmic loading control.

### Chromatin immunoprecipitation assay

We used previously described protocols [63, 64]. The following antibodies were used: anti-p53 (DO-1), anti-p65 (C-20) and IgG (sc-2025 or sc-2027) (Santa Cruz Biotechnology). ChIP-qPCR experiments were performed using Sybr MasterMix (Kapa Biosystems) and 2 μl of enriched DNA. Results were analyzed by the comparative Ct method (ΔCt) and normalized as % of input. Regions in the promoter of GAPDH or ACTB and p21 or MCP1 genes served as negative and positive controls, respectively. Primers were selected using Primer 3 (http://primer3.ut.ee/).

### Migration and wound healing assays

The migration potential of MCF7 cells was monitored by a real-time technique using the xCELLigence Instrument (Acea Biosciences, Euroclone) and CIM-16 plates, following manufacturer's instructions. Prior to the analysis, cells were grown in estrogen-free medium for two days and left untreated (mock) or treated with Doxo, TNF⍺ or the combination. 16 hours after the treatments, cells were detached and added to the top chamber in serum-free medium. Migration was detected every 10 minutes for 24 hours. We used 0.5% and 5% FBS as chemo-attractant. Migration and Invasion were also measured by QCM^TM^ Fluor 24-Well Cell Migration and Cell Invasion kits (Merck-Millipore, Milan, Italy), according to manufacturer's instructions. For wound healing, cells were seeded in 12-well plates and treated with Doxo, TNF⍺ or the combination. After 16 hours a scratch was introduced using a 10 μl pipette tip. Images of the same field were acquired immediately (T0) and after 24 hours (T24) using an automated Zeiss microscope and the AxioVision3.1 software in multidimensional mode with mosaic (3×3) acquisition.

### Flow cytometry

MCF7 cells, seeded and treated as described above, were washed with PBS and harvested by 0.05% trypsin/0.025% EDTA. The cells were washed again with PBS containing 2% FBS before being subjected to antibody binding, a combination of fluorochrome-conjugated monoclonal antibodies against human CD44 (APC) and CD24 (FITC) or their respective isotype controls (BD Biosciences, Milan, Italy) and incubated on ice in the dark for 30 minutes. Cells were then washed twice with PBS/2% FBS and resuspended in PBS. Flow cytometry analysis was conducted using a FACSCanto II instrument (BD Biosciences).

## SUPPLEMENTARY FIGURES AND TABLES








