# Supplementary material for: Cooperative interactions between p53 and NFκB enhance cell plasticity
Source: Oncotarget. 2014 Oct 21;5(23):12111–25. doi: 10.18632/oncotarget.2545 (PMC4322992; doi:10.18632/oncotarget.2545)
Supplement: Supplementary file 1 [file oncotarget-05-12111-s001.pdf]

SUPPLEMENTARY FIGURES AND TABLES

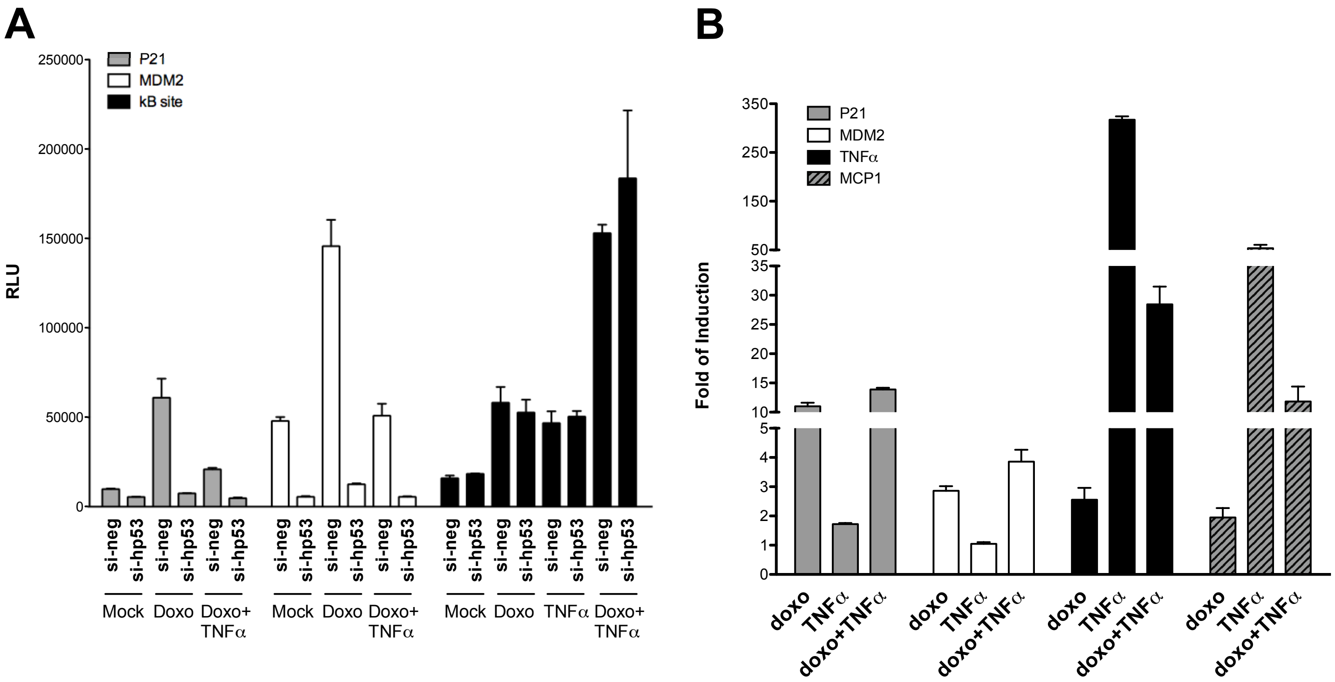

Supplementary Figure S1: Gene reporter and qPCR assays to probe p53 and p65 activity in MCF7 cells. (A) Gene reporter assays. To test p53 and NFκB responsiveness and effect of co-activation on reporter activity, MCF7 cells were seeded onto 24-well plates 24 hours before transfection. 250ng of pGL3 promoter based reporters, containing portion of p21 and MDM2 promoter (harboring p53 response elements) or a p65 responsive repeat of kB responsive sequence plasmids, were co-transfected along with 50 ng of pRL-SV40 vector, to normalize for transfection efficiency. When appropriate, 25nM of siRNA double strand oligonucleotides (Qiagen, Milan, Italy) against human p53 (si-hp53) were added to the transfection mixture. A scramble siRNA with no match in human coding RNA (25nM) was used as control (Qiagen). Cells were transiently transfected using the HighPerFect transfection reagent (Qiagen). Twenty-four hours after transfection, cells were treated with Doxo (1.5 µM) increasing concentration of TNFα (1, 5, 10 ng/ml) and with a combination of the two compounds. Sixteen hours post-treatment cells were washed with PBS and lysed with Passive Lysis Buffer 1X and luciferase activity was analyzed using the Dual-Luciferase Reporter Assay System (Promega, Milan, Italy). MCF7 cells were transiently transfected with pGL3-based reporter vectors containing fragments of the p53 responsive MDM2 intron 1, p21 promoter or a p65 responsive repeat of kB response elements. A control vector expressing the Renilla luciferase was cotransfected to normalize for transfection efficiency. Short ds-RNA oligonucleotides targeting the p53 mRNA or a scramble control were also co-transfected. 24 hours after the transfection cells were treated as indicated. Dual luciferase assays were perfomed 16 hours after the treatment. Presented in the graph are the average relative light units and the standard deviations of three biological replicates. **(B) qPCR assays**. Relative changes in the expression of the P21, MDM2, TNFα, and MCP1 genes were measured by qPCR in MCF7 cells treated with Doxo (1.5 µM), TNFα (5 ng/ml) or the combination of the two drugs. mRNA was isolated 16 hours after treatment, as described in the Methods Section. Bars plot the average fold of induction relative to the mock condition and error bars plot the standard deviations of three technical replicates. The entire experiment was repeated two times with consistent results.

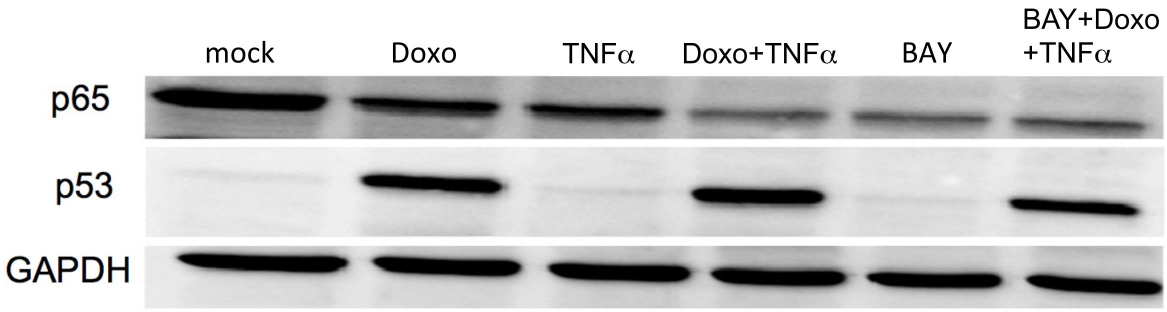

Supplementary Figure S2: p53 and p65 protein levels in MCF7 cells treated with Doxo, or/and TNFα, or/and BAY 11–7082. Immunodetection of p65, p53 and the GAPDH loading control, from total MCF7 protein extracts prepared from cell treated with the indicated molecules. See Methods for details on the antibodies used.

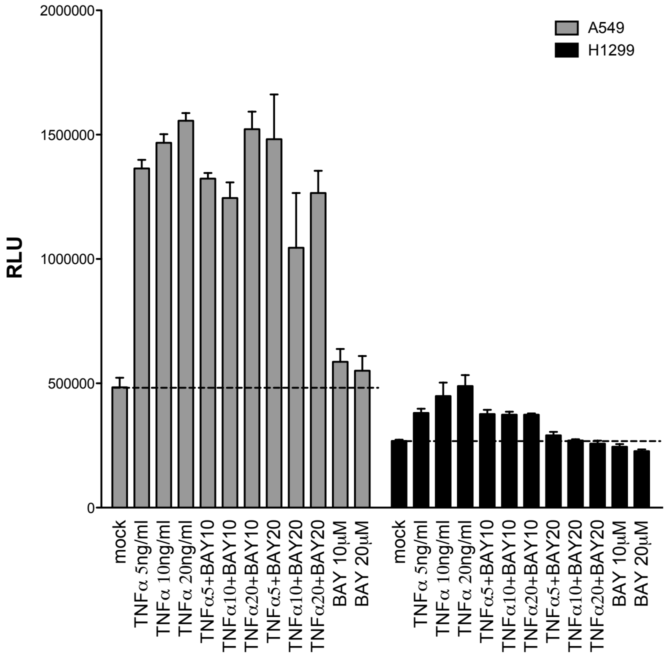

Supplementary Figure S3: Gene reporter assay in A549 and H1299 cells. The experiment was performed as described for Figure S1. The different concentrations of TNFα and BAY are indicated.

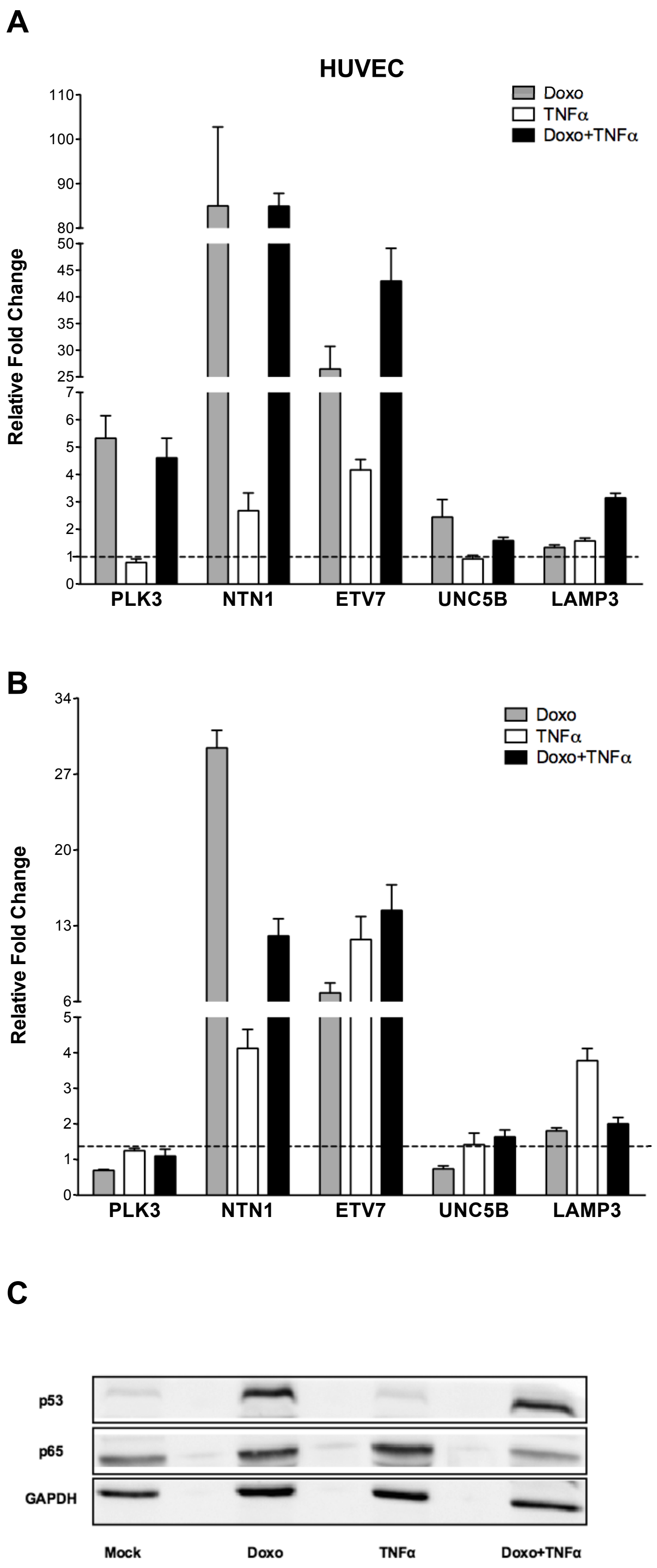

Supplementary Figure S4: PLK3, NTN1, ETV7, UNC5B and LAMP3 responsiveness varied among biological repeats in HUVEC cells. (A, B) Relative fold change expression of the indicated genes measured by qPCR. The two panels are representative of two different results each obtained in two biological replicates. **(C)** Western blot and immune-detection of p53, p65 and the GAPDH loading control.

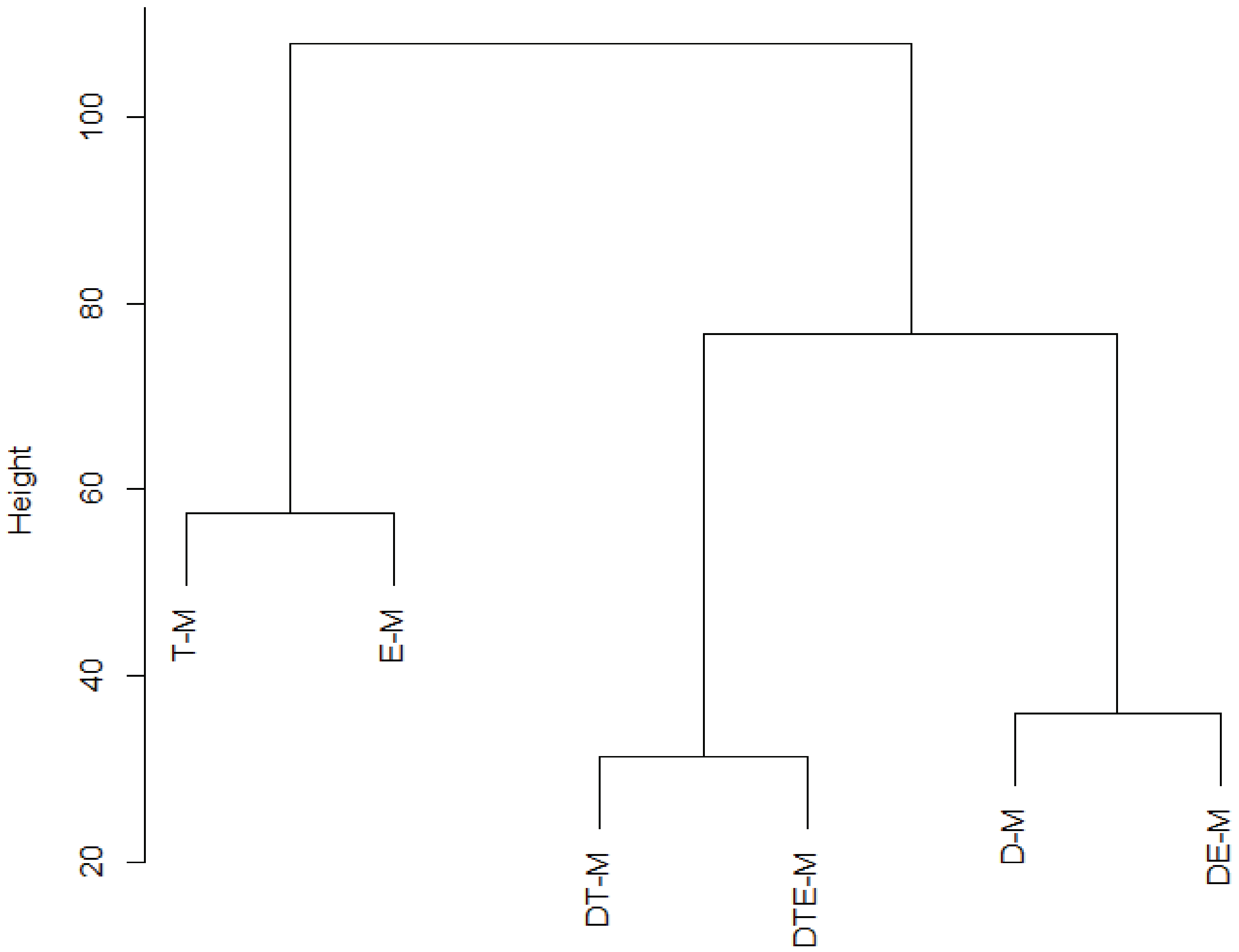

Supplementary Figure S5: Hierarchical cluster analysis. Hierarchical cluster analysis of all the treatments performed in MCF7 cells and profiled with microarrays, based on the fold changes of 1099 genes whose expression resulted to be significantly changed in at least one of the treatments (Euclidean distances and Ward clustering method). D-M (doxo vs mock); T-M (TNFα vs mock); E-M (E2 vs mock); DT-M (Doxo + TNFα vs mock); DE-M (Doxo + E2 vs mock); DTE-M (Doxo + TNFα + E2 vs mock).
